# Sex‐dependent impact of microbiota status on cerebral μ‐opioid receptor density in fischer rats

**DOI:** 10.1111/ejn.15666

**Published:** 2022-04-24

**Authors:** Felix Effah, Nívea Karla de Gusmão Taveiros Silva, Katie Vijayanathan, Rosana Camarini, Fatima Joly, Benjamin Taiwo, Sylvie Rabot, Gaëlle Champeil‐Potokar, Vincent Bombail, Alexis Bailey

**Affiliations:** ^1^ Pharmacology Section St George's University of London London UK; ^2^ Pharmacology Department Universidade de Sao Paulo São Paulo Brazil; ^3^ Université Paris‐Saclay, INRAE, AgroParisTech, Micalis Institute Jouy‐en‐Josas France; ^4^ UMR PNCA, AgroParisTech, INRAE, Université Paris‐Saclay Paris France

**Keywords:** germ‐free, microbiota, opioid receptors, rat brain, receptor ontogeny

## Abstract

μ‐opioid receptors (MOPr) play a critical role in social play, reward and pain, in a sex‐ and age‐dependent manner. There is evidence to suggest that sex and age differences in brain MOPr density may be responsible for this variability; however, little is known about the factors driving these differences in cerebral MOPr density. Emerging evidence highlights gut microbiota's critical influence and its bidirectional interaction with the brain on neurodevelopment. Therefore, we aimed to determine the impact of gut microbiota on MOPr density in male and female brains at different developmental stages. Quantitative [^3^H]DAMGO autoradiographic binding was carried out in the forebrain of male and female conventional (CON) and germ‐free (GF) rats at postnatal days (PND) 8, 22 and 116–150. Significant ‘microbiota status X sex’, ‘age X brain region’ interactions and microbiota status‐ and age‐dependent effects on MOPr binding were uncovered. Microbiota status influenced MOPr levels in males but not females, with higher MOPr levels observed in GF versus CON rats overall regions and age groups. In contrast, no overall sex differences were observed in GF or CON rats. Interestingly, within‐age planned comparison analysis conducted in frontal cortical and brain regions associated with reward revealed that this microbiota effect was restricted only to PND22 rats. Thus, this pilot study uncovers the critical sex‐dependent role of gut microbiota in regulating cerebral MOPr density, which is restricted to the sensitive developmental period of weaning. This may have implications in understanding the importance of microbiota during early development on opioid signalling and associated behaviours.

## Graphical abstract

The impact of gut microbiota on MOPr density in male and female brains at different developmental stages: Microbiota status influenced MOPr levels in males but not females, with higher MOPr levels observed in GF versus CON rats overall regions and age groups. There are no overall sex differences in GF or CON rats.

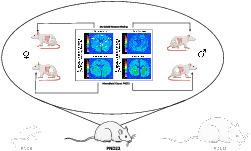

**List of Abbreviation**
AcbC: nucleus accumbens core
AcbSh: nucleus accumbens shell
AOL: anterior olfactory area‐lateral
AOM: anterior olfactory area‐medial
AOV: anterior olfactory area‐ventral
ASD: autism spectrum disorder
BCM7: beta‐casomorphin 7
Cg: cingulate cortex
CON: conventional
CPu: caudate putamen
GF: germ‐free
LOMOVO: lateral‐, medial‐, ventral‐, olfactory
MOPr: μ‐opioid receptors
M1 + M2 SUP: primary and secondary superficial motor cortex
M1 + M2 DEEP: primary and secondary deep motor cortex
M2: motor cortex
PND: postnatal days
PrL: prelimbic cortex
S1 + S2 SUP: primary and secondary superficial somatosensory cortex
S1 + S2 DEEP: deep primary and secondary somatosensory cortex
SEPTUM: Septum
TU: tubercle

## 1 INTRODUCTION

The endogenous μ‐opioid receptor (MOPr) system plays a pivotal role in mediating the effects of rewarding experiences such as social interactions and social play (Panksepp et al., 1980; Vanderschuren et al., 1995); it mediates the reinforcing properties of drugs of abuse and is a key modulator of pain and mood (le Merrer et al., 2009). Alterations of the endogenous opioid system have been linked to social functioning deficits, such as autism spectrum disorder (ASD), pain‐ and stress‐related emotional disorders and reward and reinforcement (Pellissier et al., 2018).

Age‐dependent differences in opioid sensitivity have been reported, with juveniles exhibiting higher levels of reward and sensation seeking (Smith et al., 2018) and elevated sensitivity to the social investigatory and analgesic effects of opioids compared to adults (Niikura et al., 2013; Zhang & Sweitzer, 2008). Although still under investigation, a substantial body of literature reports sex differences in opioid sensitivity, with women exhibiting higher sensitivity to pain and decreased opioid analgesic efficacy than men (Cepeda, 2003; Fillingim et al., 2009; Miller & Ernst, 2004; Mogil, 2012). In agreement, the majority of *in vivo* studies carried out in rodents consistently demonstrate a higher efficacy of morphine in modulating persistent pain in males than in females, with approximately twice as much morphine needed for females to achieve comparable levels of pain relief to males (Loyd et al., 2008; Loyd & Murphy, 2006; Posillico et al., 2015). Regarding opioid reward‐seeking behaviour, evidence suggests that, following first use, women become addicted to opiates more quickly than men (Lex, 1991; Roth et al., 2004). This increased female sensitivity towards the rewarding effects of opioids has also been reported in female rats who acquired heroin self‐administration more quickly than their male counterparts and self‐administered more significant amounts of the drug than males (Cicero et al., 2003). Factors driving these sex differences are still unclear but are believed to include a combination of genetic, hormonal and environmental factors (for a thorough review, see Becker & Chartoff, 2019).

One speculation is that differences in cerebral MOPr density may explain observed age and sex variations in opioid‐mediated behaviours. MOPr expression in the brain, like other receptors, undergoes profound species‐specific ontogenic variations throughout development (Loseth et al., 2014). In male rats, ontogenic studies revealed that MOPr are present at birth in the forebrain, including striatal reward regions, and after an initial decline in density during the first few days following birth, they undergo a subsequent rapid increase (Spain et al., 1985). Interestingly, higher levels of MOPr were recently reported in juvenile at postnatal day 35 (PND 35) male rats compared to adults (PND 84) in 11 brain regions, including the lateral septum, sub‐regions of the bed nucleus of the stria terminalis, hippocampus and thalamus (Smith et al., 2018), suggesting a brain‐specific decline of MOPr in adulthood. Given that MOPrs in these regions are known to be involved in reward processing, this increase in MOPr density has been proposed to be associated with increased susceptibility to specific social behavioural responses occurring during the juvenile period, such as reward‐driven and drug‐seeking behaviours. Unfortunately, thorough developmental studies investigating MOPr, expression variations have been mostly restricted to males, limiting our ability to understand MOPr ontogeny. Nonetheless, one study which investigated sex differences in MOPr binding in adult and juvenile rats in 33 brain regions only detected sex differences in two brain regions associated with social reward and emotionality, namely, the lateral septum and the posterior cortical nucleus of the amygdala, where higher and lower levels of MOPrs were observed in male brains compared to female, respectively (Smith et al., 2018). Whether the aforementioned differences in MOPr expression reflect distinct age‐ and sex‐related differences in opioid sensitivity discussed above, as well as the nature of the driving force behind this variability, remains to be clarified.

We hypothesize that one such driving force may be the microbial flora associated with epithelia (microbiota) or, more specifically, the gut microbiota since the gut contains most of the microbes in mammals. Emerging evidence suggests that gut microbiota plays a pivotal role in brain neurodevelopment and behaviour via the so‐called gut‐brain axis (Cryan & O'Mahony, 2011). Indeed, studies in human and animal models have identified early postnatal microbial colonization prior to weaning as critical for healthy neurodevelopment, and disruption of colonization in the susceptible periods from weaning to adolescence has been linked to disturbances in brain signalling and neuropsychiatric disorders (Warner, 2019). The gut microbiota plays a crucial role in neuro‐endocrine and neuro‐immune signalling pathways (Cryan & Dinan, 2012; Nicholson et al., 2012). The microbiota is involved in the transformation of nutrients and endogenous metabolites into neuroactive molecules such as serotonin (Yano et al., 2015), short‐chain fatty acids (Morrison & Preston, 2016), gamma‐aminobutyric acid (Matsumoto et al., 2012), indole (Mir et al., 2020) and beta‐casomorphin‐7 (Rueda‐Ruzafa et al., 2020). The latter is a milk β‐casein‐derived metabolite that possesses opioid‐like characteristics. It can bind to opioid receptors in the gut (Mohanty et al., 2016) and brain to induce gastrointestinal and behavioural effects, respectively, presumably via a gut‐brain axis mechanism (Rueda‐Ruzafa et al., 2020). Indeed, growing evidence points towards a significant influence of the opioid system through the gut microbiota‐brain axis in several neurodevelopmental neuro‐psychopathologies, including ASD, depression, anxiety and schizophrenia (for an extensive review, see Rueda‐Ruzafa et al., 2020).

Interestingly, evidence points towards a role for specific components of bacteria in modifying the expression of opioid receptors and central opioid‐mediated behaviours. For instance, it was demonstrated that some *Lactobacillus* species can induce MOPr expression in the gut and can modulate intestinal pain providing a direct and causal link between microbiota species and MOPr expression modulation, which can influence behaviour (Rousseaux et al., 2007). More recently, analgesic morphine tolerance was shown to be associated with microbial dysbiosis with selective depletion in *Bifidobacteria* and *Lactobacillaceae*, clearly linking certain gut microbiota species with central opioid‐mediated behavioural effects (Zhang et al., 2019). Nonetheless, questions remain on the impact of gut microbiota on central MOPr expressions during developmentally sensitive periods characterized by profound neuroadaptation.

Given the critical role of MOPrs in social reward, pain and emotional regulation and the evidence that gut microbiota can affect central MOPr‐mediated behaviours, we hypothesized that they are also involved in the ontogenic development of the central MOPr system. Therefore, we carried out quantitative MOPr autoradiographic binding studies on brains from germ‐free (GF) and conventional (CON) rats at different developmental ages (juvenile PND8, weaning PND22 and adult PND116‐150). In addition, due to the aforementioned sexually dimorphic nature of MOPr expression and MOPr‐mediated behaviours, we assessed the effect of gut microbiota on cerebral MOPr ontogeny in both male and female rats.

The aim of the study was twofold: first, to determine the ontogeny profile of MOPr density throughout specific developmental ages (PND 8, 22 and adult) in several cortical and non‐cortical regions of Fisher rats and to assess whether that is affected by sex, microbiota status and/or their interaction. Also, we aimed to address whether MOPr density is affected by gut microbiota status, sex or by their interaction overall brain regions and developmental ages and in specific forebrain regions and regions associated with reward.

For detailed justification of the selection of these specific age groups as well as for the selection of the GF model, rather than the antibiotic‐treated one to assess the impact of gut microbiota on MOPr ontogeny, the reader is directed to our recent publication (Effah et al., 2021) and the discussion of this manuscript.

We selected 16 brain regions for MOPr binding analysis, based on known involvement of the MOPr system in these regions in regulating social behaviour, mood, sexual behaviour and stress‐related emotional behaviours (Pasternak & Pan, 2013).

This is the first study to uncover a sex‐specific role of gut microbiota on MOPr ontogeny, highlighting the potential importance of microbiota gut health during early development on controlling central opioid receptor signalling and hence opioid‐mediated behaviours.

## 2 MATERIALS AND METHODS

### 2.1 Animals

Male and female germ‐free (GF) and conventional (CON) Fischer rats (Fischer 344; age range from 8 to 150 days old) were used. GF rats were obtained from the breeding unit of Anaxem, the GF facility of the Micalis Institute (INRAE, Jouy‐en‐Josas, France), and CON rats were descendants of three males and three females initially purchased from Charles River Laboratories (L'Arbresle, France). Following their purchase, CON rats were bred in‐house to generate enough breeding pairs and gather enough samples at the time points required for our investigation. Samples were, therefore, from the second and third generations. The fact that the Fischer 344 strain is inbred reduces the impact of any genetic variability between individuals.

All standardized procedures, including the breeding of GF animals, were carried out in France in licenced animal facilities (Anaxem license number: B78‐33‐6). To maintain axenic status, the GF rats were grown in sterile isolators, and every week, their sterile conditions were monitored by microscopic examination and screening cultures in their faeces. Makrolon cages containing sterile beddings made of wood shavings hosted the GF animals within the isolators. The CON rats were kept under a standard laboratory environment (Bombail et al., 2019). GF rats were given free access to autoclaved tap water and a gamma‐irradiated (45 kGy) standard diet (R03; Scientific Animal Food and Engineering, Augy, France). CON rats were exposed to regular tap water and the same diet (non‐irradiated). CON control rats were bred in our unit under conditions as similar as possible to the GF group: They were fed the same diet, spent the whole of their life in similar cages and bedding, were bred in house and weaned at the same age and experience the same social environment (same breeding protocol and housed in pairs from weaning). The animal room was maintained on a 12 h:12 h light:dark cycle (lights switched on at 7:30 AM to 7:30 PM). On different days, the rats were sacrificed by decapitation, and their brains were rapidly removed, frozen in isopentane and then stored at −80°C. GF and CON rat brains were processed for quantitative receptor autoradiographic analysis.

### 2.2 MOPr autoradiography

General methods for autoradiographic binding were carried out as previously described (Charbogne et al., 2017; Georgiou et al., 2015, 2016; Kliewer et al., 2019). Brains of male and female GF and CON rats at PND ages 8, 22 and 116–150 (adult) days were removed from a −80°C freezer and sectioned using a cryostat apparatus (Thermoscientific, UK) set at −21°C. Adjacent coronal brain sections of 20 μm thick cut at 400 μm intervals were thaw‐mounted onto gelatine‐coated ice‐cold microscope slides. Sections cut range from the olfactory bulb level (Bregma 4.20 mm) to the forebrain (Bregma 1.20 mm). Brain slides were conserved at −40°C in airtight containers on a layer of anhydrous calcium sulphate (Drierite‐BDH chemicals, Dorset, UK) to dry for a minimum of one week before use. Quantitative MOPr autoradiographic binding was carried out on those brain sections. Sections were rinsed for 10 min in a pre‐incubation buffer solution (50 mM Tris–HCl pH 7.4 at room temperature) to wash out endogenous opioids. Total binding was determined by incubating the prepared sections with [^3^H]DAMGO (4 nM) in an incubation buffer medium (50 mM Tris–HCl, 10 mM MgCl_2_, 1 mM EDTA, 0.1% w/v bovine serum albumin, 0.05% w/v bacitracin; Sigma‐Aldrich, Poole, UK, pH 7.4 at room temperature) for 60 min. For the non‐specific binding, adjacent sections were incubated with [^3^H] DAMGO (4 nM) in the incubation buffer in the presence of 1 μM naloxone for 60 min. When the incubation was completed, slides were rinsed three times for 5 min in ice‐cold rinse buffer solution (50 mM Tris–HCl, 10 mM MgCl_2_, pH 7.4 at 0°C) followed by a 30‐min wash in the ice‐cold rinse buffer and a subsequent 2‐s wash in ice‐cold distilled water. Slides were then dried under a stream of cool air for 2 h and stored in sealed containers with anhydrous calcium sulphate for two days. For 2 months, the slide sections were placed side by side to Kodak MR‐1 films in hyper cassettes with autoradiographic [^3^H] microscales of known radioactive concentration (Georgiou et al., 2016). Sections for the same developmental groups (i.e., CON and GF, males and females) were arranged in parallel, processed and apposed to the same film simultaneously to avoid inter‐experimental variations. Film development was conducted in a dark room using red‐filter light. The films were developed by immersing them individually one at a time into a tray containing 50% Kodak D19 developer for three minutes. The films were then immersed in a second tray containing distilled water and three glacial acetic acid solution drops for 30 s to stop the development reaction. For at least 2 min, this was followed with a fixation step by immersing the films into a third tray containing Kodak rapid fix solution. Ultimately, the films were thoroughly rinsed under cold running water for 20 min and left to dry on hanging clips in a fume hood.

### 2.3 MCID image analysis

Quantitative analysis of autoradiographic films was carried out by video‐based, computerized densitometry using an MCID image analyser as previously described by Kitchen et al. (1997). Optical density values were quantified from the [^3^H]‐microscale standards of known radioactive concentration and were entered into a calibration table on MCID. Specific binding was calculated by subtracting non‐specific binding from total binding and expressed as fmol/mg tissue equivalents. Brain structures were identified by reference to the rat atlas of Paxinos and Watson (2013). Motor cortex (M2), prelimbic cortex (PrL), lateral/medial/ventral‐olfactory cortex (LOMOVO), medial anterior olfactory (AOM), ventral anterior olfactory (AOV) and lateral anterior olfactory nucleus (AOL) were analysed from Bregma 4.20 mm. The cingulate cortex (Cg), caudate putamen (CPu), septum (SEP), nucleus accumbens core (AcbC), nucleus accumbens shell (AcbSh), superficial primary and secondary motor cortex (M1 + M2 SUP), deep primary and secondary motor cortex (M1 + M2 DEEP), superficial somatosensory cortex (S1 + S2 SUP), deep somatosensory cortex (S1 + S2 DEEP) and olfactory tubercle (TU) were analysed from Bregma 1.20 mm.

### 2.4 Data analysis for quantitative receptor

Specific radioligand binding was determined for all brain structures analysed for MOPr binding in male and female CON and GF rat (*n* = 3–4 per group, three only for day 8 CON males, GF males and females). Linear mixed model analysis with Sex, Microbiota Status, Age, Brain Region X Microbiota Status, Brain Region X Sex, Brain Region X Age, Microbiota Status X Sex, Microbiota Status X Age, Sex X Age, Brain Region X Microbiota Status X Sex, Brain Region X Microbiota Status X Age, Brain Region X Sex X Age, Microbiota Status X Sex X Age, Brain Region X Microbiota Status X Sex X Age as fixed factor variables, ‘Brain Region’ as repeated measures and rat ID as random effect factor followed by Bonferroni post hoc test corrected for multiple comparisons were performed for the determination of the effect of these factors and their two, three and four‐way interactions on MOPr binding. Bonferroni post hoc test selected to correct for type I error following multiple comparison testing was only performed if the linear mixed model revealed a significant factorial or interaction effect.

During the preparation of this manuscript, another laboratory reported elevated MOPr mRNA levels in the frontal cortex of male GF mice of 8 weeks of age (i.e., young adults) (Johnson & Burnet, 2020). Additionally, a recent report described that microbial alterations were correlated to the sensitivity to morphine reward in rats (Zhang et al., 2020), pointing towards a significant interaction between gut microbiota and the endogenous opioid reward system. Based on these studies, we hypothesized that MOPr density in the frontal cortex regions such as the anterior cingulate and motor cortex and regions associated with a reward such as the nucleus accumbens and caudate‐putamen would be particularly affected by the complete absence of microbiota in GF rats. For that reason, we decided to focus on those brain regions in particular. Therefore, we carried out planned comparison analysis of MOPr in frontal cortical and reward brain regions of male and female CON and GF rats at each of the three developmental ages to assess the effect of microbiota status and sex on MOPr density of PND 8, PND 22 and PND 116‐150 (adult) rats of these specific regions.

Linear model analysis was carried out using SPSS, and all other statistical analyses were performed using GraphPad Prism 8.

## 3 RESULTS

### 3.1 Ontogenic variation in MOPr receptor binding

Significant ‘Age,’ ‘Brain Region,’ and ‘Sex X Microbiota Status’ and ‘Brain Region X Age’ interaction effects on MOPr binding were demonstrated (Table 1). ‘Sex X Microbiota Status X Age X Brain Region’ interaction was not statistically significant (Table 1).

**TABLE 1:** Linear mixed model analysis with brain region, sex, microbiota status and age as fixed factor variables

Source	Numerator *df*	Denominator *df*	*F*	Sig.
Intercept	1	37.904	1233.135	0.000
Brain Reg	15	37.698	86.887	0.000
MICROBIOTA status	1	37.904	4.895	0.033
Sex	1	37.904	0.352	0.556
Age GRP	2	37.869	24.172	0.000
Brain Reg * GF status	15	37.698	1.490	0.159
Brain Reg * Sex	15	37.698	0.826	0.644
Brain Reg * Age GRP	30	41.521	12.480	0.000
MICROBIOTA status * Sex	1	37.904	6.445	0.015
MICROBIOTA status * Age GRP	2	37.869	2.219	0.123
Sex * Age GRP	2	37.869	2.413	0.103
Brain Reg * MICROBIOTA STATUS* Sex	15	37.698	1.668	0.101
Brain Reg * microbiota status * Age GRP	30	41.521	0.994	0.500
Brain Reg * Sex * Age GRP	30	41.521	1.032	0.456
MICROBIOTA status * Sex * Age GRP	2	37.869	0.378	0.688
Brain Reg * MICROBIOTA status * Sex * Age GRP	30	41.521	1.007	0.484

Abbreviations: GF, germ‐free; GRP, group; REG, regions.

The pairwise comparison revealed striking developmental variations of MOPr levels across all forebrain regions, sex and microbiota status groups over the first 150 days from birth (age effect, *p* < 0.001; Table 1). A significant increase in MOPr binding was detected across all regions at PND 22 versus PND 8 (*P* < 0.001), which persisted in adulthood (*p* < 0.001) (Bonferroni correction post hoc comparison; Table S1). In addition, significant developmental variations within forebrain regions were observed (Age X Region interaction, *p* < 0.001; Table 1).

**FIGURE 1:**
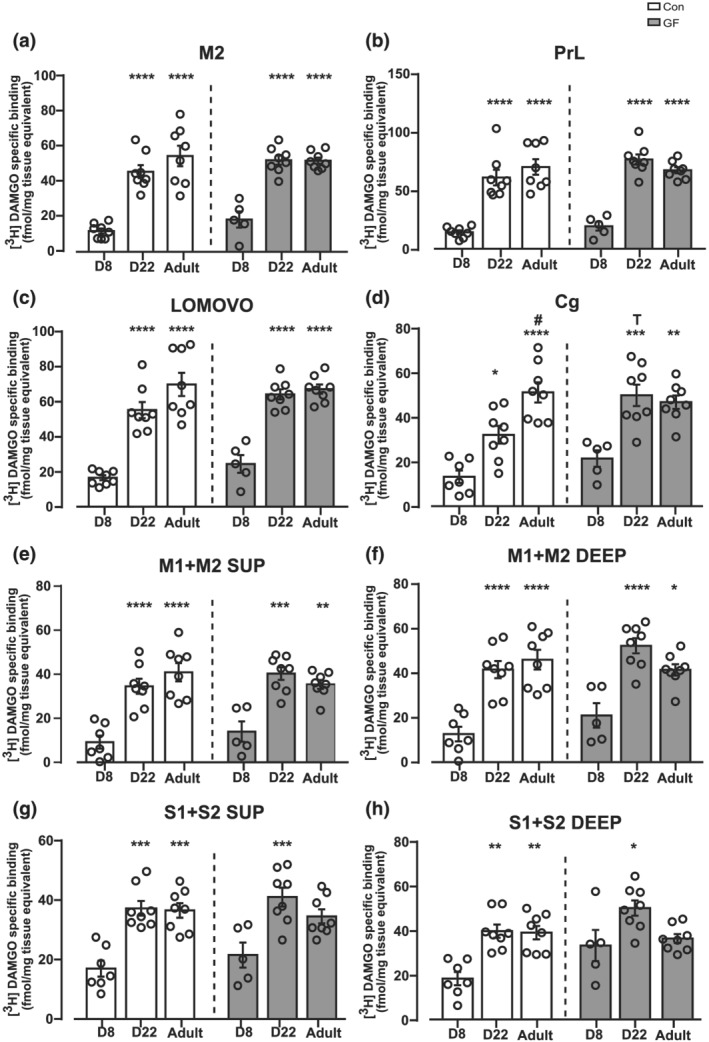
Significant ontogenic variation in MOPr binding in cortical forebrain regions in CON and GF Fischer rats. This figure illustrates [^3^H]DAMGO specific binding in cortical forebrain sections from CON and GF rats at PND 8, 22 and adult. Binding associated with male and female rats are combined in each group analysed. The concentration of [^3^H]DAMGO used for MOPr labelling was 4 nM. Quantitative MOPr binding levels are presented in the (a) M2, (b) PrL, (c) LOMOVO, (d) Cg, (e) M1 + M2 SUP, (f) M1 + M2 DEEP, (g) S1 + S2 SUP and (h) S1 + S2 DEEP. Data are expressed as mean ± S.E.M (*n* = 6–8 per group) [^3^H]DAMGO specific binding (fmol/mg tissue equivalent). *p < 0.05, **p < 0.01,***p < 0.001, ^****^ *p <* 0.0001 versus PND 8; ^#^ *p* < 0.05 vs. PND 22 within each microbiota status group, ^*T*^ *p* < 0.05 versus PND22 CON (Bonferroni post hoc analysis corrected for multiple comparisons following a linear mixed model analysis [‘brain region X age X microbiota’ interaction]). Abbreviations: M2, motor cortex; PrL, prelimbic cortex; LOMOVO, lateral‐, medial‐, ventral‐, olfactory; Cg, cingulate cortex; M1 + M2 SUP, primary and secondary superficial motor cortex; M1 + M2 DEEP, primary and secondary deep motor cortex; S1 + S2 SUP, primary and secondary superficial somatosensory cortex; S1 + S2 DEEP, deep primary and secondary somatosensory cortex

**FIGURE 2:**
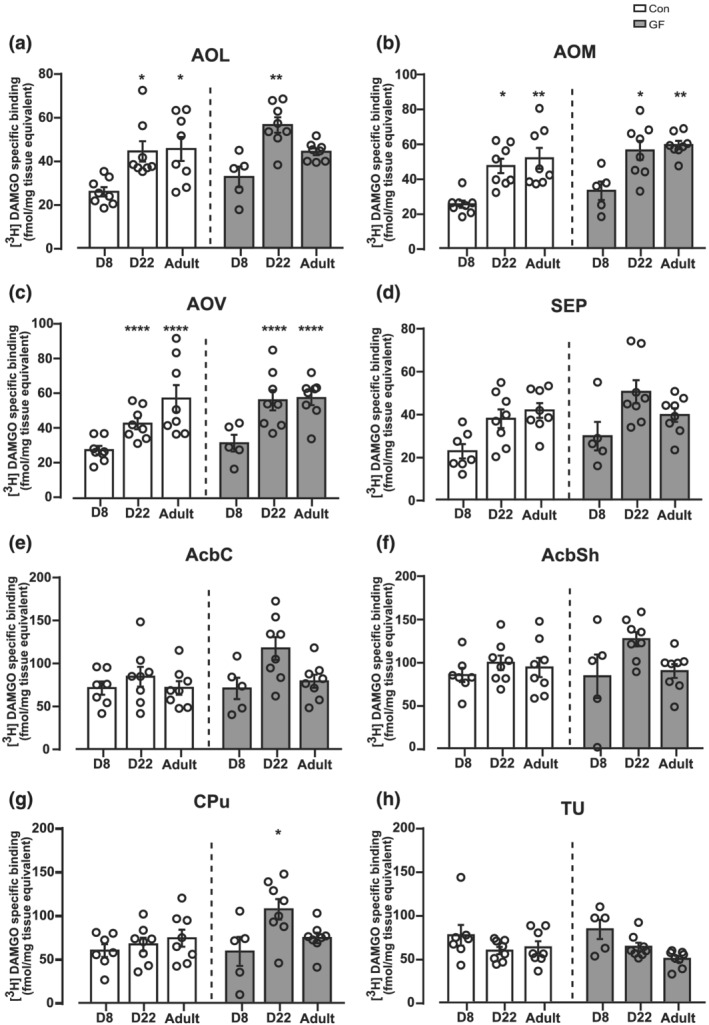
Significant ontogenic variation in MOPr binding in non‐cortical forebrain regions in CON and GF Fischer rats. This figure illustrates [^3^H]DAMGO specific binding in non‐cortical forebrain sections from CON and GF rats at PND 8, 22 and adult. Binding associated with male and female rats are combined in each group analysed. The concentration of [^3^H]DAMGO used for MOPr labelling was 4 nM. Quantitative MOPr binding levels are presented in the (a) AOL, (b) AOM, (c) AOV, (d) SEPTUM, (e) AcbC, (f) AcbSh, (g) CPu and (h) TU. Data are expressed as mean ± S.E.M (*n* = 6–8 per group) specific [^3^H]DAMGO binding (fmol/mg tissue equivalent). *p < 0.05, **p < 0.01,***p < 0.001, ^****^ *p* < 0.0001 versus PND 8 within each microbiota status group (Bonferroni post hoc analysis corrected for multiple comparisons following a linear mixed model analysis [‘brain region X age X microbiota’ interaction]). Abbreviations: AOL, anterior olfactory area‐lateral; AOM, anterior olfactory area‐medial; AOV, anterior olfactory area‐ventral; SEPTUM, septum; AcbC, nucleus accumbens core; AcbSh, nucleus accumbens shell; CPu, caudate putamen; TU, tubercle

All eight cortical regions (Figure 1) and three out of the eight non‐cortical brain regions analysed showed a significant ontogenic variation in CON rats (Figures 1 and 2). A significant twofold to fivefold increase in MOPr levels was observed in the M2, PrL, LOMOVO, AOM, AOV, AOL, Cg, M1 + M2 Sup and deep, S1 + S2 Sup and deep at PND 22 versus PND8, which persisted at the same high binding levels in adulthood (Figures 1 and 2). No significant ontogenic changes in MOPr density was observed in the Acb, Tu, Sep and CPu across the three developmental ages (*p* > 0.05) (Figure 2).

### 3.2 Effect of microbiota, sex and their interaction on MOPr binding

While no ‘Sex’ effect was identified (*P* > 0.05), significant ‘Microbiota Status’ (*P* < 0.05) and ‘Sex X Microbiota Status’ interactions (*P* < 0.05) were detected across all regions and age groups (Table 1). Pairwise microbiota status comparison across all regions, sexes and age groups revealed significantly higher levels of MOPr in GF brains compared to CON (52.9 ± 2.0 vs. 46.7 ± 2.0, *P* < 0.05). The microbiota status effect was restricted to male rats, with significantly higher MOPr binding levels detected in GF males than CON male rats across all regions and age groups (Table 2). No sex differences were observed in either GF or CON rats (Table 2).

**TABLE 2:** Linear mixed model analysis with microbiota status and sex as fixed factor variables

GF status * Sex						
Microbiota Status	Sex	Mean	Std. error	*df*	95% Confidence Interval	
					Lower Bound	Upper Bound
CON	Female	49.409	2.719	37.197	43.902	54.917
	Male	43.893	2.881	38.021	38.060	49.726
GF	Female	48.484	2.692	37.398	43.032	53.937
	Male	57.366*	3.037	38.789	51.222	63.510

Abbreviations: CON, conventional; GF, germ‐free.

* *p* < 0.05 versus male CON.

One of the key aims of this study was to assess the impact of sex, microbiota status and/or their interaction on ontogeny variations of MOPr density across three developmental stages. Based on our results (see Table 1), we conclude that neither gut microbiota (microbiota status X age interaction *p* > 0.05) nor sex (age X sex interaction *P* > 0.05) nor their interaction (gut microbiota X sex X age interaction, *p* > 0.05) influences MOPr overall regions pattern of ontogenic variation. MOPr ontogenic variations in GF rats are illustrated in Figures 1 and 2. With the exception of Cg where a statistically significant increase in MOPr was detected in GF versus CON rats only in PND 22 rats (*p* < 0.05; Figure 1), no significant changes in MOPr ontogenic variation were detected between GF and CON rats across any of the regions analysed (*p* > 0.05, Figures 1 and 2). Nonetheless, one has to point out a noticeable trend (albeit non‐significant) for higher levels of MOPr in the GF group only at PND 22 across all brain regions.

Planned comparison analysis of MOPr levels in frontal cortical and reward brain regions of male and female CON and GF rats at PND 8, 22 and adulthood.

**FIGURE 3:**
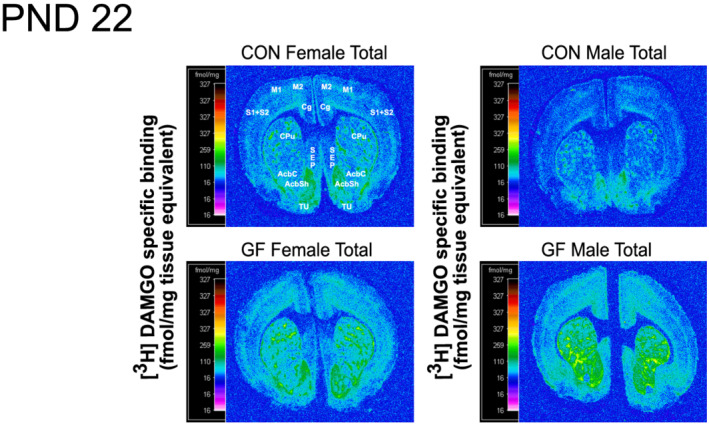
Computer‐enhanced representative autoradiograms of MOPr binding in coronal forebrain sections of male and female GF and CON rats at PND22. The represented images are of total [^3^H]DAMGO binding of coronal brain sections of PND22 at the level of the CPu and SEP (Bregma 1.20 mm). [^3^H]DAMGO (4 nM) was used for total binding. Regions analysed from this bregma have been labelled in CON females. The colour bar shows pseudo‐colour interpretation of relative density of black and white film image calibrated in fmol/mg tissue equivalent. Abbreviations: GF, germ‐free; CON, conventional; CPu, caudate putamen; SEP, septum; M1, primary motor cortex; M2, secdondary motor cortex; Cg, cingulate cortex; AcbC, nucleus accumbens core; AcbSh, nucleus accumbens shell; S1 + S2, primary and secondary somatosensory cortex; Tu, tubercle; PND, postnatal day

**FIGURE 4:**
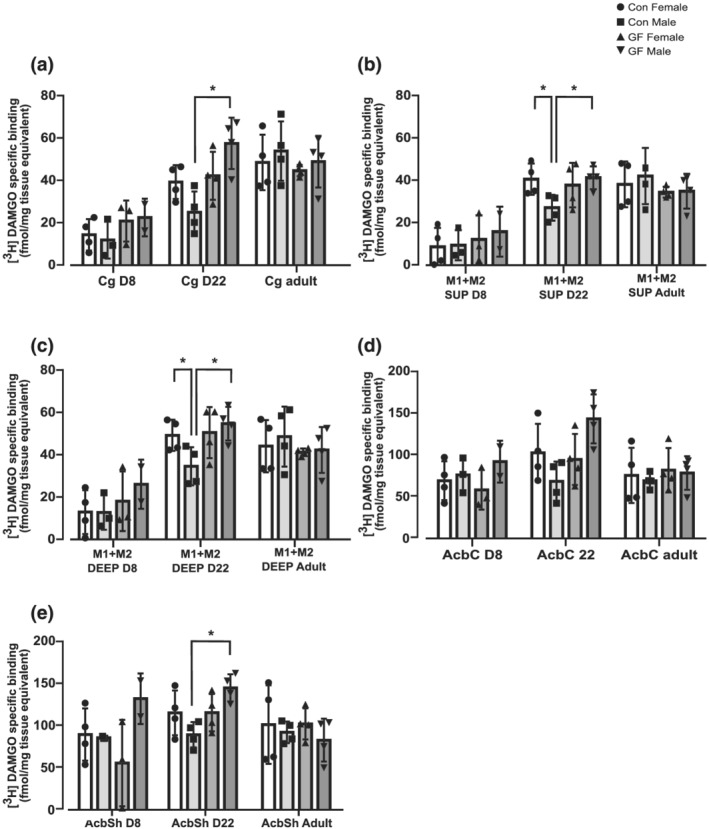
Planned comparison analysis of MOPr binding in frontal cortical and reward brain regions of male and female CON and GF Fischer rats at different developmental ages. This figure illustrates [^3^H]DAMGO specific binding in selected brain sections from female and male CON and GF rats at PND 8, 22 and adult. The concentration of [^3^H]DAMGO used for MOPr labelling was 4 nM. Quantitative MOPr binding levels are presented in the (a) Cg, (b) M1 + M2 SUP, (c) M1 + M2 DEEP, (d) AcbC and (e) AcbSh. Data are expressed as mean ± S.E.M (*n* = 3–4 per group) [^3^H]DAMGO specific binding (fmol/mg tissue equivalent). *p < 0.05 (planned comparison Bonferroni post hoc analysis corrected for multiple comparisons following two‐way ANOVA in each region and age group). Abbreviations: Cg, cingulate cortex; M1 + M2 SUP, superficial primary and secondary motor cortex; M1 + M2 DEEP, deep primary and secondary motor cortex; AcbC, nucleus accumbens core; AcbSh, nucleus accumbens shell

As discussed above, recent studies demonstrated changes in MOPr expression in the frontal cortex of young adult GF mice (Johnson & Burnet, 2020) and a correlation between microbial alterations and sensitivity to morphine reward (Zhang et al., 2020). These led us to focus our analysis on the effect of microbiota status and sex in the brain's frontal cortical and reward regions at each of the three developmental ages. Interestingly, age‐planned comparison analysis of frontal cortical and reward brain regions revealed that the aforementioned microbiota X sex effect was restricted to the nucleus accumbens shell and cingulate and motor cortex of PND22 brains (Figures 3 and 4). While in females, no ‘Microbiota Status’ differences in MOPr were detected in PND22 rats, in males, significantly higher levels of MOPr were observed in GF rats compared to CON in the Cg, AcbSh and M1 + M2(superficial and deep) (Figures 3 and 4). In the motor cortex (superficial and deep) of CON PND22 rats, while significantly lower levels of MOPr were observed in males versus females, this sex difference disappeared in the GF rats (Figure 4). No sex or microbiota status differences or their interactions were observed at PND8 or adulthood in any region analysed (Figure 4).

## 4 DISCUSSION

This study reveals a profound sex‐dependent influence of gut microbiota on MOPr density overall three developmental ages in the rat forebrain. Although mixed model linear analysis failed to reveal an effect of microbiota or sex on MOPr ontogenic variation, planned comparison analysis in the frontal cortex and reward‐associated brain regions further demonstrated that this sex‐dependent microbiota effect was restricted to PND 22, coinciding with the sensitive developmental period of weaning. To our knowledge, this is the first study to provide evidence for a critical role of microbiota on ontogenic receptor development. These findings will pave the way for future studies focusing on understanding the role of microbiota on brain development and its behavioural impact, which may have implications in the aetiology of certain neurodevelopmental disorders such as ASD.

The qualitative pattern of MOPr distribution we observed in the forebrain of both CON and GF rats is consistent with multiple autoradiographic rodent binding studies in the literature (Charbogne et al., 2017; Georgiou et al., 2015, 2016; Kliewer et al., 2019; Kornblum et al., 1987; van den Berg et al., 1999). MOPrs are present in the brain at birth and are subject to developmental plasticity thereafter (Kent et al., 1981). Interestingly, we detected a distinct pattern of ontogenic development of MOPr in the forebrain with all cortical regions and olfactory nuclei, showing a profound increase of MOPr levels from PND 8 to PND22 which was maintained through to adulthood (PND 150), and the nucleus accumbens core, Septum, Tubercle and caudate putamen showing no ontogenic variation across the three developmental ages. A similar pattern in MOPr ontogenic variation has also been detected in the forebrain of a different rat strain (Sprague–Dawley) by homogenate binding assay; after declining during the first few days after birth, MOPr binding underwent a subsequent rapid increase which peaked at PND21 and maintained high into adulthood (Spain et al., 1985).

Further studies are warranted to determine the significance of these developmental changes in MOPr on behavioural development during a sensitive developmental period. Nonetheless, our study confirms previous observations in different strains and species and expands them to assess the impact of the sex and microbiota status on the MOPr ontogenic profile. Interestingly, the lack of significant interactions between ‘age and sex’, ‘age X microbiota status’, ‘age X microbiota status X sex’ and ‘age X microbiota status X sex X region’ may signify that the ontogenic patterns of variation of MOPr, at least at those three developmental ages, may not be affected by sex or microbiota status or their interaction across and within brain regions except for Cg maybe.

Despite the large body of literature describing sex differences in opioid‐mediated behaviours, such as those involving reward and pain (Fillingim et al., 2009; Lynch & Carroll, 1999; Vathy et al., 2003; Zubieta et al., 1999), no significant sex differences were detected in CON or GF rats overall regions and developmental stages analysed. Interestingly, planned comparison analysis, specifically in the frontal cortex and reward regions, detected sex differences in CON rats only in one brain region and only one developmental time point. More specifically, we demonstrated that MOPrs are significantly higher in females compared to male rats at PND22 in the motor cortex, a region strongly associated with motivational behaviour (Galaro et al., 2019). No other sex differences were observed in either GF or CON rats in any other regions analysed. The surprisingly minor sex differences observed in our study is in agreement with a recent MOPr autoradiographic binding study which only found sex differences in 2 out of 33 rat brain regions (Smith et al., 2018), which suggest that sex differences in opioid sensitivity and opioid‐mediated behaviours are unlikely to be explained by sex differences in MOPr density.

Nonetheless, the high MOPr density detected in the motor cortex of female rats in our study is in line with PET scan studies revealing higher MOPr binding in cortical regions of women compared to men (Zubieta et al., 1999) and in several brain regions in female rats compared to males (Smith et al., 2018; Vathy et al., 2003). Whether this sex difference in the motor cortex underlines the increased sensitivity to opioid rewarding behaviours observed in females compared to males (Cicero et al., 2003; Lex, 1991; Roth et al., 2004) remains to be elucidated. However, the fact that the sex difference in MOPr was restricted to prepuberty rat brains strongly suggests that this sex effect is not driven by circulating gonadal hormones, which agrees with previous observations (Smith et al., 2018).

MOPr has a critical role in emotion processing, social behaviour and pain; there is evidence that gut microbiota can affect central MOPr‐mediated behaviours via the gut‐brain axis (GBA) (Ren & Lotfipour, 2020; Rueda‐Ruzafa et al., 2020). Therefore, we investigated in GF rats, the impact of microbiota on the expression of MOPr in forebrain regions at different developmental periods, including early life, where significant neuroadaptations are known to occur. A significant ‘Microbiota Status’ and ‘Sex X Microbiota Status’ interaction effect was detected in over‐all regions, and age groups with higher levels of MOPr observed in GF brains compared to CON. Interestingly, the microbiota status effect was restricted to male rats, with significantly higher levels of MOPr binding detected in GF compared to CON male rats across all regions and age groups. In addition, planned comparison analysis in the frontal cortex and reward brain regions further demonstrated that this sex‐dependent microbiota effect was detected in the cingulate, motor cortex and nucleus accumbens shell of PND 22 but not PND8 or adult rats. This is in agreement with a recent report published independently from our laboratory reporting elevated MOPr mRNA levels in the frontal cortex of male GF versus CON mice of 8 weeks of age (Johnson & Burnet, 2020). Our study goes beyond the Johnson and Burnet (2020) study demonstrating a correlation of mRNA level changes with the more physiological relevant receptor binding changes and extends the investigation to both males and females and rats of different developmental ages. Moreover, these observations suggest that this microbiota effect on MOPr might be conserved across different rodent species.

Given that Johnson and Burnet (2020) reported higher MOPr expression (mRNA levels) in GF mice, the enhanced levels of MOPr binding detected in male GF rats in our study is likely to reflect an increase in MOPr transcription. However, this upregulation may also be driven by differences in endogenous opioid peptide levels in the forebrain of GF rats compared to CON, as lower levels of *Pomc* (the gene that encodes for beta‐endorphin) expression was detected in the frontal cortex of germ‐free mice (Johnson & Burnet, 2020). As such, this MOPr upregulation may reflect a homeostatic compensatory mechanism to reduce beta‐endorphin levels in the forebrain. Indeed, there is ample evidence that beta‐endorphin exposure induces MOPr internalization and blockade of MOPr causes compensatory upregulation of MOPr levels (Bailey et al., 2003; Lesscher et al., 2003; Yabaluri & Medzihradsky, 1997). Alternatively, high levels of MOPr activation may lead to inhibition of pro‐opiomelanocortin neuron activity, resulting in lower concentrations of the peptide, as demonstrated by Pennock et al. (2012).

Our study clearly demonstrates that the presence of microbiota in male CON rats modulates central MOPr levels. GF rats' complete absence of microbiota upregulates the receptors, suggesting a profound role of gut microbiota in regulating cerebral function MOPr. These findings add to the growing literature demonstrating a pivotal role for gut microbiota on brain neurodevelopment which may impact behaviour and performance (Dinan & Cryan, 2016; Warner, 2019) and extend it to the central MOPr system. Interestingly, manipulation of specific microbiota strains has been associated with alteration in peripheral MOPr expression and central opioid‐mediated behaviours. For example, Rousseaux et al. (2007) reported that a *Lactobacillus reuteri*, *a* probiotic strain, can alter MOPr expression in the gut and can modulate intestinal pain. Analgesic morphine tolerance was shown to be associated with microbial dysbiosis with selective depletion in *Bifidobacteria* and *Lactobacillaceae* (Zhang et al., 2019). Thus, future work should focus on identifying the nature of the microbiota species that contribute to the regulation of central MOPrs and opioid‐mediated behaviours to develop novel therapeutic strategies for disorders characterized by deregulation of the opioid system.

Interestingly, at least in frontal cortical and reward regions, the impact of microbiota in our study was restricted to PND 22 rats which correspond to the age when weaning takes place in rats. Weaning is a critical developmental process that involves the dietary transition from breast milk to solid food. This nutritional event coincides with physiological and psychological adjustments and changes to social interactions. Thus, it is possible that the profound impact of microbiota at this specific sensitive stage of development on MOPr density may be reflective of a microbiota X nutrition interaction effect on MOPr development. As discussed above, milk contains the milk β‐casein, which gets degraded by host and gut microbial enzymes to an active opioid peptide (exorphin) beta‐casomorphin 7 (BCM7) with a high affinity for μ‐opioid receptors (MOPr). At the same time, *Bifidobacterium* seems to have the ability to degrade BCM7 (Sakurai et al., 2018). High levels of circulating BCM7 have been linked to neurodevelopmental disorders, including ASD (Sokolov et al., 2014). It is unknown whether the lack of microbiota in GF rats affects the circulating levels of BCM7 and whether that affects MOPr density and behaviour. Nonetheless, given the impact of gut microbiota on BCM7 production coupled with defects that GF rodents exhibit in the gut (Round & Mazmanian, 2009; Smith et al., 2007) and blood–brain barrier permeability (Braniste et al., 2014), it is likely that circulation BCM7 may be altered, thus contributing to changes in central MOPr activation and neurodevelopmental defects in the social domain. The fact that GF rodents exhibit behaviours reminiscent of ASD, such as a reduced preference for social interactions and social novelty (Buffington et al., 2016; Desbonnet et al., 2014; Luk et al., 2018; Stilling et al., 2018), as well as repetitive behaviours (Desbonnet et al., 2014) is supportive of such hypothesis which nonetheless warrants future investigation.

The microbiota effect on MOPr binding was restricted to male rats, and the presence of sex differences in the microbiome‐gut‐brain axis is in agreement with multiple studies (Coretti et al., 2017; Davis et al., 2017; Leclercq et al., 2017; Sylvia et al., 2017). This sex‐dependent microbiota effect is consistent with our recent findings demonstrating an identical oxytocin receptor density regulation pattern in male GF rats (Effah et al., 2021). The mechanism underlining the increased sensitivity of microbiota regulation of MOPr in males and its resistance in females remains to be elucidated. However, it is unlikely to reflect changes in circulating gonadal hormone levels at least at PND 22, as these are at undetectable levels at weaning age. Instead, they may be caused by sex‐specific differences in gut microbiota profiles (Coretti et al., 2017) or by sex‐specific epigenetic regulation of genes (Keiser & Wood, 2019). Future research should investigate the potential behavioural consequence of this sex difference of microbiota effect on central MOPr to determine its role in the aetiology and development of neurodevelopmental disorders such as ASD, which is higher among males. Intriguingly, consistent with our observation with MOPr and OTR regulation, GF male mice show more pronounced social impairments (Hoban et al., 2016) and alterations in neurochemistry (Clarke et al., 2013) compared to females, which has led to the hypothesis that sex differences in gut microbiota may reflect the higher incidence of neurodevelopmental disorders such as ASD among males.

Interestingly, we detected that brain regions where sex‐specific upregulation of MOPr in the GF group was detected in PND 22 included the nucleus accumbens shell, a key reward region of the brain, suggesting a potential sex difference on opioid reward behaviour driven by microbiota. Whether this reflects a sex difference on the impact of gut microbiota on reward behaviour remains to be elucidated. However, given the role of MOPr in social reward behaviour and ASD (Becker et al., 2014), especially in the period immediately after weaning, this may be a conceivable explanation. As pups grow away from their mother and the litter, weaning age corresponds to a period of enrichment and expansion of the social environment, achieved through play. Play behaviour constitutes an opportunity to ‘train for the unexpected’ (Spinka et al., 2001) as juveniles learn valuable physical and social skills, and the endogenous opioid system has been causally linked to the rewarding effects of social play (Trezza et al., 2011). Morphine treatment has been reported to enhance the pleasurable and motivational aspects of rat playful behaviour (Achterberg et al., 2019).

One ought to point out the limitations of this study. The low sample number of rats allocated to each age, sex, microbiota status group resulted in lower statistical power, which may lie behind the lack of significant four‐way (sex X age X microbiota status X region) interactions. As such, this study may be considered as a pilot study. Furthermore, this current work is limited by the restricted availability of germ‐free strains. Given the role of MOPr in drug‐seeking behaviour (le Merrer et al., 2009), it would be intriguing to assess the impact of microbiota status in Lewis rats which are characterized with much higher responsivity to drugs of abuse, including heroin compared to Fischer rats which are far less responsive (Picetti et al., 2012). Nonetheless, there are no Lewis GF lines to our knowledge.

Another potential limitation that one should consider is the inherent differences in genetics, birth and weaning/housing environment and diet between CON and GF rats. Nonetheless, and in line with previous studies (Bercik et al., 2011; Chevalier et al., 2020; Erny et al., 2015; Gareau et al., 2011), CON control rats were bred in our unit under conditions as similar as possible to the GF group: They were fed the same diet, spent the whole of their life in similar cages and bedding, were bred in house and weaned at the same age and experience the same social environment (same breeding protocol and housed in pairs from weaning). Hence, it is unlikely that the changes in MOPr observed are due to confounding factors associated with diet and environment.

GF rats were chosen as the preferable animal model to compare against conventional rats for this study for several reasons. This study aimed to unravel the impact of the absence of gut microbiota on brain development and, more specifically, on the MOPr system's ontogeny. Therefore, the GF model is the most suitable animal model to achieve this aim. An alternative to the GF model is the antibiotic‐treated model. This model is obtained as a result of antibiotic cocktail administration to the rats, which broadly deplete their gut microbiota. However, this method is incapable of depleting the gut microbiota thoroughly (Kennedy et al., 2018), and therefore, there may be some bacteria still present that could have impacted the outcome of this study. Had antibiotic‐treated models been used for this investigation, it would have been difficult to determine at what developmental stage the absence of the gut microbiota initiates impact on brain MOPr neurochemistry. In addition, since rats are highly susceptible to antibiotic‐induced diarrhoea, had this happened, it could have caused a change in mood and may have led to alterations in the central MOPr expression in the rats. Although not an ideal model to investigate the phenotypical impact of MOPr regulation such as drug addiction, our choice of the Fischer rat line was limited by the availability of strains with GF status. Nonetheless, the fact that Fischer rats are inbred would reduce the risk of confounder genetic variability.

Given the current evidence pointing towards a key role for the microbiota in mediating opioid‐mediated behaviours, it would be intriguing to assess the behavioural impact of the sex‐dependent microbiota effects detected in our study and is thus warranted. Behavioural tests carried out in GF rats of the same colony as the ones used in our study revealed an increased anxiogenic and stress‐reactive phenotype than conventional rats (Crumeyrolle‐Arias et al., 2014; Jaglin et al., 2018) and lower dopaminergic turnover rate in brain regions involved in stress regulation and reward including the frontal cortex, hippocampus and striatum (Crumeyrolle‐Arias et al., 2014). Whether these phenotypes reflect sex‐dependent microbiota effects on MOPr detected in our study remains to be determined.

In conclusion, this study uncovers for the first time a key sex‐dependent role of gut microbiota in regulating MOPr levels in the brain, which is restricted to the sensitive developmental period of weaning, at least in the frontal cortex and nucleus accumbens shell. This highlights the importance of gut microbiota health during early development on opioid signalling in the brain and possibly regulating opioid‐mediated behaviours such as a social reward. In addition, this may raise concern over the risk of the development of neuropsychopathologies characterized by alterations in MOPr brain signalling such as ASD following severe disturbances of gut microbiota at an early developmental stage.

## CONFLICT OF INTEREST

The authors declare no conflict of interest.

## AUTHOR CONTRIBUTIONS

Felix Effah carried out the experiments, data acquisition and data analysis; contributed to writing the manuscript and produced the first draft of the paper. Nivea Karla de Gusmao Taveiros Silva carried out the data analysis, preparation of figures and tables and contributed to the writing of the manuscript. Katie Vijayanathan contributed to the data acquisition and analysis. Rosana – contributed to the study design, data analysis and interpretation and written part of the manuscript. Fatima Joly bred the animals used in this study and contributed to the study design. Benjamin Taiwo contributed to the writing of the manuscript. Sylvie Rabot bred the animals used in this study and contributed to the data interpretation and writing of the manuscript. Gaëlle Champeil‐Potokar contributed to experimental work. Vincent Bombail is the correspondent author who conceived the study, planned study design, contributed to experimental work and contributed to the manuscript's data analysis, interpretation and writing. Alexis Bailey is the correspondent author who conceived and planned the study design and contributed to the manuscript's data analysis, data interpretation and writing.

### PEER REVIEW

The peer review history for this article is available at https://publons.com/publon/10.1111/ejn.15666.

## Supporting information




**Table S1.** Linear mixed model analysis revealing the effect of age on MOPr binding across the three age groups.

## Data Availability

All data generated or analysed during this study are included in this published article (and its supporting information)
