# Supplementary material for: Sex‐dependent impact of microbiota status on cerebral μ‐opioid receptor density in fischer rats
Source: Eur J Neurosci. 2022 Apr 24;55(8):1917–33. doi: 10.1111/ejn.15666 (PMC9324823; doi:10.1111/ejn.15666)
Supplement: Supplementary file 1 — Table S1. Linear mixed model analysis revealing the effect of age on MOPr binding across the three age groups. [file EJN-55-1917-s001.docx]

**Supplementary Table 1**. Linear mixed model analysis revealing the effect of age on MOPr binding across the three age groups.

|  |  | Age GRP |  |  |  |
| --- | --- | --- | --- | --- | --- |
|  |  |  |  | 95% Confidence | Interval |
| Age GRP | Mean | Std. Error | df | Lower bound | Upper bound |
| PND 8 | 35.573 | 2.576 | 39.171 | 30.363 | 40.782 |
| PND 22 | 58.070*** | 2.393 | 37.197 | 53.221 | 62.918 |
| Adult | 55.722*** | 2.393 | 37.197 | 50.874 | 60.571 |

Abbreviations: GRP, group; PND 8, postnatal day 8; PND 22, postnatal day 22. ***P<0.001 vs. PND8
